# Towards a common approach for managing food allergy and serious allergic reactions (anaphylaxis) at school. GA^2^LEN and EFA consensus statement

**DOI:** 10.1002/clt2.70013

**Published:** 2024-12-29

**Authors:** Antoine Deschildre, Montserrat Alvaro‐Lozano, Antonella Muraro, Marcia Podesta, Debra de Silva, Mattia Giovannini, Simona Barni, Timothy E. Dribin, Mónica Sandoval‐Ruballos, Aikaterini Anagnostou, Alessandro Fiocchi, Alice Toniolo, Andrew Bird, Angel Sánchez Sanz, Anna Asarnoj, Anna Nowak‐Wegrzyn, Berber Vlieg‐Boerstra, Brian P. Vickery, Carina Venter, Caroline Nilsson, Cecilia Parente, Céline Demoulin, David M. Fleischer, Diola Bijlhout, Edward F. Knol, Eleanor Garrow, Emma E. Cook, Fallon Schultz, Francesca Lazzarotto, Francesca Mori, Gary Wong, Gideon Lack, Graham Roberts, Gustavo Andres Marino, H. N. G. Oude Elberink, Helen A. Brough, Hugh A. Sampson, Jay Lieberman, Jennifer Gerdts, Jing Zhao, Josefine Gradman, Julia E. M. Upton, Julie Wang, Kati Palosuo, Kirsi M. Järvinen, Kirsten Beyer, Kunling Shen, Laura Polloni, Lianne Mandelbaum, Luciana Kase Tanno, Lucy A. Bilaver, Marcus S. Shaker, Margitta Worm, Maria Said, Mary Kelly, Mary Jane Marchisotto, Michael Makris, Mikaela Odemyr, Montserrat Fernandez‐Rivas, Motohiro Ebisawa, Nandinee Patel, Pablo Rodríguez del Río, Pakit Vichyanond, Paul Turner, Pete Smith, Pilar Morón Gaspar, R. Sharon Chinthrajah, Rima Rachid, Roberta Bonaguro, Ruchi Gupta, Sabine Schnadt, Sakura Sato, Stefania Arasi, Stephanie Leonard, Sung Poblete, Susanne Halken, Thuy‐My Le, Guillaume Pouessel, Tracey Dunn, Victoria Cardona, Torsten Zuberbier

**Affiliations:** ^1^ University Lille Lille France; ^2^ CHU Lille Lille France; ^3^ Hôpital Jeanne de Flandre Lille France; ^4^ Hospital Sant Joan de Déu Barcelona Spain; ^5^ Universitat de Barcelona Barcelona Spain; ^6^ Padua University Hospital Padua Italy; ^7^ GA2LEN Berlin Germany; ^8^ European Federation of Allergy and Airways Diseases Patients' Associations Brussels Belgium; ^9^ Food Allergy Italia Padova Italy; ^10^ The Evidence Centre London UK; ^11^ Meyer Children's Hospital IRCCS Florence Italy; ^12^ University of Florence Florence Italy; ^13^ Meyer Children's Hospital IRCCS Florence Italy; ^14^ Cincinnati Children's Hospital Medical Center Cincinnati Ohio USA; ^15^ University of Cincinnati Cincinnati Ohio USA; ^16^ Hospital Sant Joan de Déu Barcelona Spain; ^17^ Children's Hospital Houston Texas USA; ^18^ Baylor College of Medicine Houston Texas USA; ^19^ Pediatric Hospital Bambino Gesù Rome Italy; ^20^ Padua University Hospital Padua Italy; ^21^ University of Texas Southwestern University Dallas Texas USA; ^22^ AEPNAA Spanish Association of People with Food and Latex Allergy Madrid Spain; ^23^ Karolinska Institutet Stockholm Sweden; ^24^ Astrid Lindgren Children's Hospital Stockholm Sweden; ^25^ Hassenfeld Children's Hospital New York New York USA; ^26^ University of Warmia and Mazury Olsztyn Poland; ^27^ OLVG Hospital Amsterdam the Netherlands; ^28^ Rijnstate Hospital Arnhem the Netherlands; ^29^ Emory University School of Medicine and Children's Healthcare of Atlanta Atlanta Georgia USA; ^30^ Children's Hospital Colorado Denver Colorado USA; ^31^ Karolinska Institutet Stockholm Sweden; ^32^ Sachs' Children and Youth Hospital Stockholm Sweden; ^33^ Hospital Italiano de Buenos Aires Buenos Aires Argentina; ^34^ AFPRAL Paris France; ^35^ University of Colorado School of Medicine Aurora Colorado USA; ^36^ Children's Hospital Colorado Denver Colorado USA; ^37^ University of Wales Trinity St David London UK; ^38^ University Medical Center Utrecht Utrecht The Netherlands; ^39^ Food Allergy and Anaphylaxis Connection Team West Chester Ohio USA; ^40^ Hokkaido University Hokkaido Japan; ^41^ ATOPICCO Network for Children of the Earth Tokyo Japan; ^42^ International FPIES Association Quincy Massachusetts USA; ^43^ Padua University Hospital Padua Italy; ^44^ Meyer Children's Hospital IRCCS Florence Italy; ^45^ Chinese University of Hong Kong Hong Kong China; ^46^ Guy's and St Thomas' NHS Foundation Trust London UK; ^47^ King's College London London UK; ^48^ University of Southampton Southampton UK; ^49^ St Mary's Hospital Southampton UK; ^50^ NIHR Biomedical Research Centre Southampton UK; ^51^ University Hospital Southampton NHS Foundation Trust Southampton UK; ^52^ Hospital Universitario Austral and Fundación SOS Alergia Pilar Argentina; ^53^ University of Groningen Groningen The Netherlands; ^54^ Guy's and St Thomas' NHS Foundation Trust London UK; ^55^ King's College London London UK; ^56^ Icahn School of Medicine at Mount Sinai New York New York USA; ^57^ University of Tennessee Health Science Center Memphis Tennessee USA; ^58^ LeBonheur Children's Hospital Memphis Tennessee USA; ^59^ Food Allergy Canada North York Ontario Canada; ^60^ Capital Institute of Pediatrics Affiliated Children Hospital Beijing China; ^61^ Hans Christian Andersen Children's Hospital Odense Denmark; ^62^ University of Toronto Toronto Ontario Canada; ^63^ Icahn School of Medicine at Mount Sinai New York New York USA; ^64^ Helsinki University Hospital Helsinki Finland; ^65^ University of Rochester New York New York USA; ^66^ Charité Universitätsmedizin Berlin Berlin Germany; ^67^ Shenzhen Children's Hospital Beijing China; ^68^ Beijing Children's Hospital Beijing China; ^69^ Padua University Hospital Padua Italy; ^70^ No Nut Traveler Inc Livingston New Jersey USA; ^71^ Hôpital Arnaud de Villeneuve ‐ University Hospital of Montpellier Montpellier France; ^72^ Northwestern University Evanston Illinois USA; ^73^ Geisel School of Medicine at Dartmouth Hanover New Hampshire USA; ^74^ Dartmouth‐Hitchcock Medical Center Lebanon New Hampshire USA; ^75^ Charite Univeritätsmedizin Berlin Berlin Germany; ^76^ Allergy & Anaphylaxis Australia Sydney New South Wales Australia; ^77^ Anaphylaxis UK Farnborough UK; ^78^ MJM Advisory LLC New York New York USA; ^79^ National and Kapodistrian University of Athens Athens Greece; ^80^ Astma‐ och Allergiförbundet Stockholm Sweden; ^81^ Hospital Clinico San Carlos Madrid Spain; ^82^ Universidad Complutense IdISSC Madrid Spain; ^83^ NHO Sagamihara National Hospital Sagamihara Japan; ^84^ Imperial College London London UK; ^85^ Hospital Infantil Universitario Niño Jesús Madrid Spain; ^86^ Samitivej Allergy Institute Bangkok Thailand; ^87^ Imperial College London London UK; ^88^ Griffith University Southport Queensland Australia; ^89^ AEPNAA Spanish Association of People with Food and Latex Allergy Madrid Spain; ^90^ Stanford University Stanford California USA; ^91^ Boston Children's Hospital Harvard Medical School Boston Massachusetts USA; ^92^ Padua University Hospital Padua Italy; ^93^ Northwestern University Evanston Illinois USA; ^94^ Ann & Robert H. Lurie Children's Hospital of Chicago Chicago Illinois USA; ^95^ Deutscher Allergie‐ und Asthmabund North Rhine‐Westphalia Germany; ^96^ NHO Sagamihara National Hospital Sagamihara Japan; ^97^ Bambino Gesù Children's Hospital Rome Italy; ^98^ University of California San Diego San Diego California USA; ^99^ Food Allergy Research & Education (FARE) McLean Virginia USA; ^100^ Hans Christian Andersen Children's Hospital Odense Denmark; ^101^ University of Southern Denmark Odense Denmark; ^102^ University Utrecht Utrecht The Netherlands; ^103^ Roubaix Hospital Lille France; ^104^ Lille University Hospital Lille France; ^105^ Anaphylaxis UK Farnborough UK; ^106^ Hospital Universitari Vall d'Hebron Barcelona Spain; ^107^ Charité Universitätsmedizin Berlin Berlin Germany; ^108^ Fraunhofer Institute for Translational Medicine and Pharmacology ITMP Immunology and Allergology Berlin Germany

**Keywords:** anaphylaxis, food allergy, health education, long‐term management, school health service

## Abstract

GA^2^LEN and EFA propose minimum specifications for all industrialised countries/regions to work towards to support students with food allergies in educational settings. We reviewed research and legislation and gained feedback from over 100 patient and professional groups. We built shared expectations around: 1. training all school staff about what food allergy is, the symptoms of allergic reactions, what to do in an emergency, and when and how to use and store devices that laypeople can use to administer adrenaline (epinephrine). 2. preventing allergic reactions by using clear labelling on school menus and prepacked and non‐prepacked foods and regular cleaning where students eat. 3. preparing for serious allergic reactions, with written emergency action plans for every student with food allergies, legislation allowing schools to store adrenaline for anyone who needs it in an emergency (not just those prescribed it), and training and legal safeguards for staff administering adrenaline. 4. including affected students by discussing food allergy in the curriculum, raising awareness among all students and caregivers and reviewing school processes regularly. It is time for national and international action at the policy level. Patient groups, education networks and professional societies all play a role in campaigning for shared next steps.

## 1 WHY FOCUS ON SCHOOLS?

The Global Allergy and Asthma European Network's (GA^2^LEN) Anaphylaxis and Food Allergy network (ANAcare) includes over 40 research and clinical centres specialising in allergic disease. ANAcare and the European Federation of Allergy and Airways Diseases Patients' Associations (EFA) built a consensus about immediate steps that countries/regions can take to support school students with food allergies. We use the term ‘school’ throughout, but the principles also apply to other educational settings such as childcare, kindergarten, college and university.

It is essential to have international agreement about a common approach in schools because:

- Growing numbers of school‐aged people are being diagnosed with food allergies.^1^, ^2^, ^3^ The number of students experiencing a serious allergic reaction (anaphylaxis) is also increasing.^4^
- Everyone with food allergies needs to manage their exposure to allergens carefully, including at school. Students spend at least one third of their time at school. Up to one third of food‐allergic students may experience an allergic reaction at school, and many of these students have not previously been diagnosed with food allergies.^5^ Students often experience a serious allergic reaction for the first time at school.^6^
- Serious allergic reactions to food can be distressing and frightening. Symptoms may include swelling of the lips and tongue, difficulty breathing, rash or itchy skin, dizziness or fainting, vomiting and diarrhoea.^7^ It is rare to die from a serious allergic reaction to food, but around 1 in 5 school‐age people who die from allergic reactions are exposed to the food allergen at school.^8^, ^9^
- Even as they gain independence, students rely on school staff to keep them safe from food allergens.^10^ However, a large proportion of school staff say they do not know enough about managing food allergies and do not feel confident in helping a child experiencing a serious allergic reaction.^11^
- Some countries/regions have developed valuable guidelines, training and legislation to help manage food allergies in schools, but this is not consistent worldwide. Even when guidelines and resources exist, they may not be applied well in practice. Within the same country or even city, individual schools may have widely varying policies and different staff skills around food allergy.
- Guidelines recommend using adrenaline (also called epinephrine) as the first‐line treatment for serious allergic reactions when warranted because symptoms can escalate rapidly.^12^, ^13^ There are significant differences between countries in whether schools are allowed to stock supplies of adrenaline for this purpose and in legal safeguards for school staff administering adrenaline.^14^ This inconsistency can lead to confusion and fear about what school staff should do in an emergency.
- Students with food allergies can feel isolated, be excluded from activities such as cooking classes and class trips and be bullied for being ‘different’. Schools may not always know how to create an inclusive environment that welcomes students with their conditions and requirements.

Guidelines, systematic reviews and position papers have highlighted the importance of managing food allergy well at school.^15^, ^16^ Yet, how best to do so remains unclear. This consensus statement moves the conversation forward. It is unique because it describes agreements from over 100 stakeholder organisations across more than 20 countries.

Having a common framework is a step towards more equity among countries/regions, schools and affected students. Processes to keep students safe should not be a ‘postcode lottery’, with stark variations depending on where they live and the local economic context. We hope that setting out expectations based on good practice will help countries/regions consider how to update the local approach, particularly in areas where national policies do not currently exist.

We aim to make this framework accessible to lawmakers, school staff, patient groups and professionals who are not necessarily allergy specialists. We have written this statement in an easy‐to‐understand style, without technical terms. For example, we use the phrase ‘serious allergic reaction’ throughout instead of the technical term anaphylaxis.

## 2 HOW DID WE REACH A CONSENSUS?

A multidisciplinary working group with patient organisations, paediatricians, allergy specialists, school staff, primary care staff and researchers oversaw the process to build consensus around key steps to best manage food allergy in schools.

First, we identified current practices and gaps by reviewing the literature available via MEDLINE and EMBASE and by reading existing guidelines and policies. We then searched online for descriptions of local practice and legal frameworks in industrialised countries.

We contacted a sample of patient organisations to understand national and regional laws for managing food allergies at school. To our knowledge, this is the first time that anyone has systematically audited information about policies across such a wide range of countries from a patient perspective. We asked patient organisations because they are often part of governmental or national committees. Many work alongside other stakeholders to draft emergency action plan templates and deliver training so that they are aware of what is happening at a national or regional level.

We invited organisations from 26 countries with large and active patient organisations to contribute in summer 2023. 21 of them took part in interviews or completed a written template, a response rate of 81%. The countries involved from the WHO European region were Denmark, France, Germany, Greece, Italy, Poland, Portugal, Serbia, Spain, Sweden, Switzerland, Turkey and the UK. Non‐European countries involved were Argentina, Australia, Brazil, Canada, Chile, China, Japan, and the United States.

The multidisciplinary working group draughted preliminary suggestions for a common approach based on research and guidelines, patient organisation interviews and stakeholder feedback. Over a year, we asked over 100 organisations, representing thousands of stakeholders, to test and adapt our preliminary ideas. We hosted discussions at conferences, GA^2^LEN ANAcare and EFA meetings and training programmes. We sought feedback in writing. Finally, over 80 international experts validated the proposed principles by checking their feasibility and applicability in their local networks. The panel included patient organisations, education representatives, allergy specialists, paediatricians, psychologists, researchers and others from Europe, North America, South America, Australasia and East Asia. We also consulted with the Association for Teacher Education in Europe and local groups working closely with schools. There was near universal consensus about the principles to include in a common approach, so a Delphi process was not needed.

## 3 WHAT APPROACH COULD COUNTRIES TAKE?

Table 1 presents GA2LEN and EFA's shared position around the key areas that countries and regions could focus on to better manage food allergy in schools, namely:

1. increasing the knowledge and confidence of school staff
2. preventing allergic reactions to food
3. planning when and how to respond in an emergency, including access to approved adrenaline administration devices for serious allergic reactions
4. building wider awareness and an inclusive school culture to reduce isolation and bullying

**TABLE 1:** Proposed common approach to better manage food allergy in schools.

Focus area	Examples of tools/procedures	Target group
1. Increase knowledge and confidence of school staff		
Assess the education needs of school staff	Structured questionnaire to assess staff awareness and confidence managing food allergies and serious allergic reactions	Entire school staff including managers, teachers, catering teams, cleaners, administrative staff, school nurses, substitute teachers, teachers in training
Train staff, using experts such as patient organisations	Workshops or online modules to increase staff confidence about their role in managing food allergies and preventing and responding to serious allergic reactions, including practical scenarios/case stories. Content to include: What food allergies are, how common they are and who is at risk Contents of food products, food labelling and risk of cross‐contamination How to reduce the risk of accidental exposure to food allergens Symptoms of allergic reactions, including serious allergic reactions, which age groups are at higher risk and why Staff role in prevention and management Importance of rapid response to serious allergic reactions When to call for help and/or activate emergency services How and when to use approved adrenaline administration devices Where adrenaline administration devices are stored Psycho‐socio‐emotional aspects of food allergy and strategies to promote inclusion and prevent bullying and risk‐taking	
2. Focus on prevention		
Use clear policies for areas where lunch/food is eaten	Cleaning schedule for tables and equipment in areas where food is eaten Clear labelling of food allergens on school menus and prepacked and non‐prepacked foods available at school Ways of identifying students with food allergy without singling them out as ‘different’ (which can impact on bullying) No school‐wide food bans/restrictions Considering whether or not policies are needed around seating arrangements and supervision, particularly when students are not developmentally ready to manage their food allergy	School management and catering teams; families
Implement laws about reasonable adjustments	Legislation that recognises food allergy as a condition or disability and mandates that schools must make reasonable adjustments to accommodate students with food allergies	Lawmakers/government authorities
3. Plan how to respond to emergencies		
Implement consistent emergency action plans, updated regularly to reflect best practice	Schools to get an up‐to‐date written emergency action plan for every child with diagnosed food allergy, which includes a standardised list of content areas Process to ensure staff are aware of what emergency action plans are for, contents of plans, where they are stored and when they need to be updated, including staff involved in before and after school activities	School management, teachers and families
Ensure approved adrenaline administration devices are available	Policies so schools can stock adrenaline administration devices for use by anyone affected, not just named individuals, and replace them prior to expiry Easy‐to‐access safe storage space (at appropriate temperature) at school and on excursions, close to students (e.g. classrooms), with staff aware of location Legal safeguards for school staff who need to administer adrenaline in an emergency (and training in how and when to use adrenaline, as above)	Lawmakers/government authorities, school management
Emergency care	Process for calling emergency services and accessing emergency care.	School staff
4. Build a positive school culture		
Improve awareness of food allergy in the school population	Age‐appropriate posters, leaflets, talks, interactive games etc. to help students and caregivers understand what food allergy is and what to do if they see someone having an allergic reaction Processes so affected students are included in activities alongside others to reduce isolation and bullying (e.g. during birthday celebrations and parties at school; safe participation in school trips and external activities)	All school students, families, teachers, government
Review school policies and processes regularly	Timetable for regular review of policies and processes with a named responsible coordinator Process to ensure that all school staff take part in training as part of their induction and have regular refresher sessions for example, every 1–2 years Process to ask students about ways to keep improving school approaches	School management, lawmakers/government authorities

The following sections provide our rationale for each of these focus areas. We have not included actions around food allergens in non‐food school supplies (such as casein in chalk) but are aware that there is work to be done here.

We aimed to develop minimum expectations that every country/region could adapt to local needs and ways of working, not to suggest a ‘one size fits all’ approach or to mandate specific policies. Our suggested minimum specifications are targeted towards the resources and legal frameworks in industrialised countries, but may also work as a model elsewhere.

Good progress is already underway in some parts of the world. If all countries worked towards a common approach with these minimum specifications, we could ensure more parity and safety for students, no matter where they live. Based on our review of national policies, there is scope to continue developing in every country and region. A common approach will also help schools respond to the new landscape emerging to support students with food allergies, including new ways to administer adrenaline for serious allergic reactions (e.g., adrenaline administered as a nasal spray).

### 3.1 Increasing knowledge across school staff

Our proposed common approach aligns with the components identified in practice guidelines (see Table 2). However, this consensus statement is more specific about increasing skills and awareness across school teams.

**TABLE 2:** Examples of guideline recommendations about managing food allergy in schools.

Canada‐led: international practice guidelines 2021^5^	Europe‐led: EAACI/GA^2^LEN task force 2010^17^	Europe‐led: EAACI 2022^18^	Australia: ASCIA 2015^19^
Training			
Schools should implement training for school staff.	School training should involve all staff members and include allergen avoidance, recognition and treatment of anaphylaxis. Staff should be aware of the location of emergency kits and expiration dates.		Staff training focussing on recognition and management of acute allergic reactions.
Prevention			
	Schools should be notified by parents or doctors when a child is identified as being at risk. All protective measures should continue during extracurricular activities.		Implementation of practical strategies to reduce accidental exposure.
Emergencies			
Schools should require all parents of students with food allergy to provide an up‐to‐date emergency action plan. Schools should implement site‐wide protocols to manage reactions in students without emergency action plans on file. Staff should administer adrenaline in an emergency only when a serious allergic reaction is suspected. Adrenaline should not be administered pre‐emptively where no signs of an allergic reaction have occurred. If the legislation allows, schools should stock general‐use adrenaline autoinjectors on‐site.	School staff should be indemnified against prosecution for the consequences of administering medication. An emergency action plan developed by allergist should be adopted. Individually labelled emergency kits should be provided for schools Trained and competent school staff should administer emergency medication to young children. Older students should be allowed to self‐medicate when sufficiently mature and trained.		Up‐to‐date ASCIA action plan available Consider the institutional provision of adrenaline autoinjectors for general use.
Culture			
Schools should not prohibit specific foods site‐wide or establish allergen‐restricted zones, although some high‐risk situations may need a different approach and restrictions may be merited when students are not developmentally ready to manage their own allergies.	If a severe reaction occurs within the school, the headmaster should take responsibility for undertaking or delegating and supervising investigation of the root cause.	School policies should reflect anaphylaxis guidelines Target secondary schools and community settings with education support.	Raise awareness that reactions may occur for the first time outside the home in those without prior food allergy diagnosis. Age‐appropriate education of students and their peers.

It is essential that school staff have the knowledge, confidence and tools to support students with food allergies and can recognise an allergic reaction and react promptly. In many parts of the world, school staff say they need more knowledge and skills.^20^, ^21^, ^22^

For example, a multi‐country study found large regional variations in the proportion of school staff who received training about food allergy or serious allergic reactions (ranging from 14% to 64%). Only 6 out of 10 staff in Italian schools, 5 out of 10 in US schools and 3 out of 10 in Spanish schools said they had ever been trained about food allergy.^23^ Another study found that only one‐quarter of teachers in Turkey would provide first aid if a child was having a serious allergic reaction. None identified that adrenaline should be used.^24^ In Spain, about one‐third of the staff were not aware of where adrenaline administration devices were located at their school.^25^

Given how widely knowledge varies, individual self‐assessment scales could help to identify gaps in the knowledge and confidence of school staff to allow more tailored training.^26^

We propose that multidisciplinary training for school staff should describe food allergy, how schools can reduce exposure to food allergens, how to identify a serious allergic reaction, how to respond rapidly and appropriately to support a student who is having an allergic reaction and how to promote inclusion and reduce bullying. This requires much more than simply training people to administer adrenaline in an emergency.^27^ Training in preventive measures could help to reduce allergic reactions.

Research suggests that training can improve staff knowledge and management of food allergies, with some studies finding benefits can be maintained for around 2 years.^28^, ^29^, ^30^, ^31^, ^32^ Some countries or regions prefer annual training to account for changes in protocols or new adrenaline administration devices. Others prefer training every 2–3 years to better fit with other mandatory training cycles. We advocate for training at least every 1–2 years.

There is limited evidence about the most effective training methods. In‐person workshops allow trainers to use scenarios, respond to questions and practice using approved devices. However, providing workshops in every school or educational district would be resource intensive. Another approach is to use pre‐recorded online modules with quizzes at the end to test comprehension. This can be done relatively inexpensively but may have variable success in terms of engagement and impact, so we do not advocate this as the main approach. It may be feasible to run live online educational sessions for several schools at once, which would have the benefit of staff being able to ask questions and learn about implementation in other schools.

Our audit across 21 countries found examples of patient organisations and partners developing standardised training to be run nationally, but this is not available consistently. We suggest that every country should follow this approach, drawing on existing initiatives to consider the ‘must have’ content to include in training. It is important that there is a schedule to refresh training regularly so that staff can maintain their skills, knowledge and confidence. A mix of theoretical training and opportunities to practice emergency response skills could be repeated at least every 1–2 years and included alongside other first aid or similar training.

Toolkits are available to support staff education.^33^ We call on stakeholder organisations to work with school districts and policymakers to consider embedding staff training as part of business as usual. More work is needed at a strategic level to consider the best ways to do this, who should lead it and how to engage with the right stakeholders.

Some staff roles may need more in‐depth training than others, but every member of school teams should receive some basic training because such a large number of students are affected and children are in contact with multiple staff, not just teachers. This would include managers and administrators, school nurses (where applicable), teachers and auxiliary staff, substitute or supply teachers, teachers in training, lunchroom and catering staff, cleaners, school transportation staff and any others who may have a student with a food allergy in their care.

### 3.2 Focussing on prevention

Schools can do much to help students minimise exposure to food allergens and prevent serious allergic reactions.^34^, ^35^ Our review of policies and laws in 21 countries found significant variations in prevention approaches (Table 3). This corresponds to other research that identified limited nationally or regionally agreed policies.^36^ In some countries, this reflects jurisdictional responsibilities because education laws are made at the regional or provincial level. However, even recognising this, stakeholders in this consensus process emphasised the need for greater consistency in implementing preventative strategies, both across schools in local areas and across countries/regions.

**TABLE 3:** National or regional policies/laws implemented for managing food allergy in schools.

	Policies about training school staff	Policies for where students eat	Policies for food in classrooms	Food policies for extra‐curricular activities	Policies for food in transport to/from school	Template for individual emergency action plan	Adrenaline autoinjectors for named students at school	Policies for stocking unassigned autoinjectors
Examples from the European continent and countries								
Denmark	‐	‐	‐	‐	‐	‐	√	‐
France	‐	√	√	√	‐	√	√	M, H
Germany	√	√	‐	‐	‐	√	√	‐
Greece	‐	‐	‐	‐	‐	‐	√	‐
Italy	√	√	√	√	√	√	√	‐
Poland	√	‐	‐	‐	‐	√	√	‐
Portugal	‐	‐	‐	‐	‐	‐	√	√ in schools with 1000+
Serbia	‐	‐	‐	‐	‐	‐	√	‐
Spain	‐	√	‐	‐	‐	‐	‐	‐
Sweden	‐	√	√	‐	‐	‐	√	‐
Switzerland	‐	√	‐	‐	‐	√	√	H
Turkey	√	√	‐	‐	‐	‐	√	‐
UK	√	√	‐	‐	√	√	√	√ except N
Examples from other countries								
Argentina	‐	‐	‐	‐	‐	‐	‐	‐
Australia	√	√	√	√	√	√	√	√
Brazil	√	√	‐	‐	‐	‐	AAI not in country	
Canada	√	‐	√	‐	‐	√	√	√
Chile	‐	‐	‐	‐	‐	‐	‐	‐
China	‐	‐	‐	‐	‐	‐	‐	‐
Japan	√	√	N	‐	‐	‐	√	‐
USA	√	√	√	√	√	√	√	√

*Source*: Patient organisations reported on national or regional policies in their countries. √ = national or widespread regional policy/law exists for some aspects, though may not be comprehensive; ‐ = no or few national/regional mandated policies/laws or wide variation, including regional/provincial guidance for schools to adapt to the local context; N = in nursery; M = in middle school; H = in high school. More details are in the online supplement.

This does not mean that every country or region needs to do exactly the same thing. Instead, we propose that every country/region should consider this set of principles and ensure that they address each area in ways that work in their local context. The important thing is not to leave individual schools to use different approaches, documents and training.

For example, steps must be taken to prevent allergic reactions to food in school catering, including during purchasing, storing, preparing, cooking, distributing and consuming.^37^ Better labelling of food content and regulated allergens on packaged and unpackaged food is essential. There is a need for more consistency in training staff to respond to emergencies during after‐school activities and travel to and from school, and having adrenaline available in these settings.

Regions and schools have varying approaches to whether students with food allergies are asked to eat in designated areas or included alongside other students. There are also differing policies about restricting some foods. We do not propose designated eating areas and food restrictions as part of our minimum expectations. Being asked to eat away from others can be stigmatising. Food restrictions (bans) may be difficult to implement, ineffective and lead to resentment amongst other students. Designated eating areas or restrictions may be useful in a small number of cases, such as for students who are developmentally reliant on others to help manage their allergies, but this should not be a universal policy.^38^

It would be helpful to have more consistent laws around reasonable adjustments that schools are expected to make for students with food allergies. Some countries have laws that protect people with disabilities from discrimination and recognise food allergies as a ‘disability’. This means that schools need to make reasonable adjustments and cannot legally deny the student's right to some protections. Other countries/regions do not have these legal protections or do not include food allergy within them. This leaves students with limited options if a school declines a family's request for reasonable adjustments.

Allergy specialists should support preventative efforts by including expectations for schools in the emergency action plans of individual students. This may include information about foods that could trigger a reaction; how to use precautionary allergen labelling, including children with food allergies in school activities according to their individual requirements; and what emergency kit students should have available and where it should be stored.

### 3.3 Planning for emergencies

Serious allergic reactions to food are more likely in schools than in any other community location.^39^ Guidelines recommend using adrenaline quickly as a first‐line treatment when warranted because symptoms can escalate rapidly.^40^, ^41^ There are several devices for administering adrenaline currently available or coming onto the market. The most common are adrenaline autoinjectors, which are pre‐dosed single‐use devices designed to be stored at room temperature. They can be used by people without specific health knowledge, qualifications, or skills. Adrenaline autoinjectors are not available in all countries. Pre‐filled syringes are one alternative.^42^

In some countries, school staff (excluding nurses) are not allowed to administer some types of medication. Serious allergic reactions are an exception because they are medical emergencies which can be life‐threatening. In most countries, staff are expected to call emergency services for help and administer adrenaline promptly if needed while waiting for emergency medical assistance. Schools execute a treatment plan based on written instructions from the student's doctor (letter/medical certificate) and an emergency action plan expressly authorised by the parents or guardians.^43^ In some countries, everyone who receives adrenaline is expected to be transported to hospital. In other countries, this is not the case.^44^, ^45^, ^46^

As part of our consensus process, stakeholders around the world suggested the need for more consistent laws regarding adrenaline use in schools. Table 3 and the supplementary online material show that there are significant differences between countries/regions in whether schools can legally store and use adrenaline in emergencies. In some cases, students are not allowed to carry their prescribed medications with them. Sometimes schools are able to hold the devices prescribed for named students. In other countries, schools can also hold stocks of devices that are not assigned for use with a specific student. These ‘unassigned’ or ‘stock’ devices can be used by anyone in an emergency, although some countries require parental consent prior to use.

Studies suggest that implementing legislation increases access to adrenaline and the use of emergency action plans in schools, consistent with good practice guidelines.^47^, ^48^, ^49^, ^50^ For example, provinces in Canada have laws requiring schools to have policies around serious allergic reactions and to hold emergency action plans for each student. In some provinces, there is provision for schools to stock unassigned adrenaline administration devices. In general, schools in areas that introduced such laws were more compliant with good practice, had higher staff awareness and administered adrenaline more effectively.^51^

Most countries do not have national policies around storing unassigned or stock adrenaline devices or find it challenging to implement them.^52^ Some countries have legal variations depending on the province or region. In areas without laws, decisions are made at the schools' discretion, depending on the child's age and the size, location and type of school.

We call for legislation which encourages schools to hold unassigned adrenaline devices. Laws should specify the number of devices to be stored, who pays for them, where they should be stored (temperature and location) and whether it is appropriate to use adrenaline prescribed for a named student for someone else in an emergency. More consistent legislation would ensure that students and staff have access to medication in an emergency, regardless of whether they have been diagnosed with a food allergy (because the first reaction could occur at school). Studies have found that school staff may also need adrenaline themselves.^53^ Schools should ask about diagnosed and suspected allergies when registering any new student and staff and have clear policies for managing serious allergic reactions in diagnosed and undiagnosed people.

It is also important that schools keep adrenaline administration devices in close proximity to the child, such as in a classroom, rather than in a medical room or administrative office. It is essential that these devices are available on field trips and out of school activities.

We also propose that schools request that each diagnosed student's family provide a written individual emergency action plan, as is legally required in some countries/regions. These personalised documents are completed and signed by healthcare professionals. They describe when and how to treat an allergic reaction. They contain a person's allergies, symptoms to watch for, what to do in the case of an allergic reaction and emergency contact information. They are designed to be shared with stakeholders such as schools and reviewed by physicians every 1–2 years. Research suggests that following such a plan can reduce the risks associated with allergic reactions.^54^

Guidelines suggest that schools should hold and be familiar with an allergic student's emergency action plan. However, the proportion of schools with copies of emergency action plans varies greatly, from 6% to 90% in different studies.^55^

Patient organisations have worked with national scientific societies or food allergy referral centres to create written emergency action plan templates. Table 4 provides examples of the content included. There are many other examples available. There is scope to harmonise the content to be included in an emergency action plan template at the national or international level. Countries/regions could then brand this and adapt to local laws and needs, but the same core headings would be included. It is also essential for schools to have a consistent procedure for using unassigned stock adrenaline for symptoms of a serious allergic reaction where a student does not have an emergency action plan.

**TABLE 4:** Content in small selection of emergency action plans.

Content included	Australia: ASCIA 2025^56^	Canada 2022^57^	France: SFA 2020^58^	Germany: DAAB 2018^59^	Italy: Food allergy Italia 2018^60^	UK: BSACI 2018^61^	US: AAAAI 2020^62^	US: AAP 2017^63^
Allergen triggers	√	√	√	√	√	√	√	√
Risk factors		Asthma; prior anaphylaxis		Asthma	Asthma; prior anaphylaxis		Asthma	Asthma; prior anaphylaxis
Whether child may carry/self‐administer medication								√
Location of adrenaline autoinjector		√		√	√			
Autoinjector expiration date		√						
Includes parent signed consent		√	√	√	√	√		√
Date of plan review	√		√					
Management of mild to moderate allergic reactions	√		√	√	√	√		√
List of symptoms of serious allergic reactions	√	√	√	√	√	√	√	√
Pictorial instructions for using autoinjector	√	√	√	√	√	√		
Positioning	Lay flat, if breathing is difficult sit with legs outstretched			Sit if breathing issues, lie down if circulatory problems; recovery position if unconscious	Lie flat with legs raised; sit if breathing issues; recovery position if unconscious	Lie flat with legs raised; sit if breathing issues		Lie on back; lie on side if vomiting or breathing issues
Adrenaline administration site	Mid outer‐thigh	Mid outer‐thigh	Mid outer‐thigh	Laterally into the thigh muscle	Outside of the thigh	Mid outer‐thigh	Thigh	
Indicates that anaphylaxis may occur without skin symptoms	√					√		
Dose of adrenaline by child's weight	0.10 mg if 7.5 kg to <13 kg; 0.15 mg if 13 kg to <25 kg, 0.3 mg if >25 kg	Specific prescribed dose	0.15 mg if <25 kg, 0.3 mg if >25 kg, 0.5 mg if teenager >60 kg	Specific prescribed dose	Specific prescribed dose	Specific prescribed dose	0.1 mg if 16.5–33 lbs, 0.15 mg if 33–66 lbs, 0.3 mg if >66 lbs.	0.1 mg if <13 kg, 0.15 mg if 13 kg to <25 kg, 0.3 mg if >25 kg Intranasal: 2 mg for >30 kg
Additional adrenaline dose after 5 min if no improvement	√	√			√	√		√
Time of observation after reaction	At least 4 h	At least 4–6 h			At least 4 h			
Advises calling for emergency help or transfer to medical centre	√	√		√	√	√	√	√

*Note*: AAAAI, American Academy of Allergy, Asthma and Immunology; AAP, American Academy of Paediatrics; ASCIA, Australasian Society of Clinical Immunology and Allergy; BSACI, British Society for Allergy and Clinical Immunology; DAAB, Deutscher Allergie‐und Asthmabund e.V.; SFA, Société Française d'Allergologie.

Targeted approaches may be needed to improve school preparedness for known high risk groups. For example, students with learning difficulties or developmental challenges need extra precautions. In some parts of the world, high schools are less likely than elementary and middle schools to hold emergency action plans even though adolescents are at greater risk of serious allergic reactions to food.^64^ It is also challenging to manage food allergies in daycare or preschool settings since very young children may be less able to identify allergens and communicate symptoms. In some places, a high proportion of those caring for young children are from different cultural backgrounds and may not speak the language fluently, which creates further barriers for training.

### 3.4 Building a positive culture

Improving the awareness of school staff can have a positive impact on students' quality of life, psychological wellbeing and safety.^11^, ^65^ However, lasting change requires raising awareness of food allergy among the entire school community, including all students and parents. This may increase inclusion and reduce the bullying and isolation that some students with food allergy experience.^66^ Schools can request a list of ‘treat’ foods that an affected student tolerates so that these foods can be used during celebrations.

One‐third of students experiencing a serious allergic reaction to food at school have not previously been diagnosed with food allergy, so a wider range of students must be aware of what to look out for and how to react.^67^ Awareness raising approaches may include posters in classrooms and lunchrooms, paragraphs in school newsletters emailed to parents and talks in class as part of the curriculum. Countries/regions could encourage schools to have a food allergy awareness day during International Food Allergy Awareness Week/Day, World Food Safety Day or similar national awareness days/weeks/months.^68^

## 4 WHAT NEXT?

Increasing the knowledge and confidence of school staff is crucial for helping students manage food allergies, but it is only part of the solution. GA^2^LEN and EFA's agree that training programmes are most effective when implemented in a supportive wider context. This includes having consistent policies and laws across countries to create shared expectations around managing food allergies in schools, including legislation that supports easy access to adrenaline.

Most guidelines and studies published in the last 2 decades suggest similar priorities, but there remain wide variations in practice.^69^, ^70^, ^71^ It is time to concentrate on practical steps to implement a common approach. This requires action at the national policy level and cross‐country collaborations. It is about having minimum specifications that all countries and regions strive for, but with scope to adapt to reflect the local legal context, needs and ways of working.

GA^2^LEN and EFA will be campaigning together for the following next steps:

- bringing together an international working group to plan the best ways to raise awareness of food allergy issues and gain buy‐in from national stakeholders
- an awareness‐raising campaign about food allergy issues in schools, including school events during International Food Allergy Awareness Week/Day
- drawing together stakeholders within each country or region to plan next steps, such as government education and health departments, patient and caregiver groups, school management, school catering organisations, political leaders, school health staff, scientific and health societies, communication experts and allergy and medical specialists
- agreeing common content/headings for an individual emergency action plan and online training sessions for school staff that can be adapted across countries by patient and professional organisations
- improving training about food allergy in undergraduate and postgraduate teacher training, placements for trainee teachers, health professional training and courses for school managers
- considering potential common principles to deal with food allergens in non‐food school supplies. For example, some chalks include casein, a protein found in milk, which could trigger an allergic reaction if an affected student inhales chalk dust

There are pressing gaps that make this challenging.^72^ Research and development is particularly needed in these areas:

- understanding how best to fund work to develop and implement a common approach across schools and countries. Cost‐benefit analyses would make the case that investing in shared policies and protocols would provide a good return on investment for quality of life. There is also potential for economic partners in regions such as the European Union to consider common pricing expectations around adrenaline administration devices
- developing effective methods to train and refresh school staff about how to prevent, recognise and treat allergic reactions to food. Patient organisations and clinical centres of excellence could provide standardised videos, modules and information sheets. Content may need to be tailored to account for the differences in school staff educational backgrounds and familiarity with food allergy
- evaluating the impact of interventions. Our literature review identified some promising research on ways to support school staff, but the studies were largely small‐scale. It is imperative to evaluate the impact of interventions on health outcomes over time. Without robust registries and mandatory reporting on the use of adrenaline devices in school settings, it is challenging to identify reliable data to monitor impacts.^73^ We suggest mandating that schools report information about symptoms, therapies and health outcomes to existing registries. This may be challenging to implement but is a potential goal to work towards
- improving access to adrenaline. Stocking unassigned adrenaline administration devices is cost‐effective in many settings^74^, ^75^ so stakeholders should encourage consistent legislation about this in schools. For example, European health legislation could encourage member states to allow schools to stock unassigned autoinjectors, similar to US laws. There is significant variation in access to adrenaline administration devices globally, so countries need to develop economical, feasible and sustainable programmes for their contexts. There is also potential to develop technology so that school staff can access real‐time guidance about whether to administer adrenaline based on patient‐specific symptoms and whether repeat adrenaline is warranted if symptoms are persistent or worsening. This would help to reduce the burden on school staff in emergencies

Optimising the prevention and management of allergic reactions to food in schools requires collaboration from families and students, school staff, medical professionals and regulators.

We call for stakeholders to work at a national and international level to implement a consistent approach so that students are free to pursue their education in a safer and more inclusive environment.

## AUTHOR CONTRIBUTIONS

**Antoine Deschildre**: Investigation; validation; writing—review and editing. **Montserrat Alvaro‐Lozano**: Writing—review and editing; validation. **Antonella Muraro**: Conceptualization; funding acquisition; writing—review and editing; supervision. **Marcia Podesta**: Methodology; writing—review and editing; investigation. **Debra de Silva**: Writing—original draft; writing—review and editing; methodology; project administration; conceptualization. **Mattia Giovannini**: Writing—review and editing; validation. **Simona Barni**: Writing—review and editing. **Timothy E. Dribin**: Writing—review and editing. **Mónica Sandoval‐Ruballos Hospital**: Writing—review and editing. **Aikaterini Anagnostou**: Writing—review and editing. **Alessandro Fiocchi**: Writing—review and editing. **Alice Toniolo**: Writing—review and editing. **Andrew Bird**: Writing—review and editing. **Angel Sánchez Sanz**: Writing—review and editing. **Anna Asarnoj**: Writing—review and editing. **Anna Nowak‐Wegrzyn**: Writing—review and editing. **Berber Vlieg‐Boerstra**: Writing—review and editing. **Brian P. Vickery**: Writing—review and editing. **Carina Venter**: Writing—review and editing. **Caroline Nilsson**: Writing—review and editing. **Cecilia Parente**: Writing—review and editing. **Céline Demoulin**: Writing—review and editing. **David M. Fleischer**: Writing—review and editing. **Diola Bijlhout**: Writing—review and editing. **Edward F. Knol**: Writing—review and editing. **Eleanor Garrow**: Writing—review and editing. **Emma E. Cook**: Writing—review and editing. **Fallon Schultz**: Writing—review and editing. **Francesca Lazzarotto**: Writing—review and editing. **Francesca Mori**: Writing—review and editing. **Gary Wong**: Writing—review and editing. **Gideon Lack**: Writing—review and editing. **Graham Roberts**: Writing—review and editing. **Gustavo Andres Marino**: Writing—review and editing. **H.N.G. Oude Elberink**: Writing—review and editing. **Helen A. Brough**: Writing—review and editing. **Hugh A. Sampson**: Writing—review and editing. **Jay Lieberman**: Writing—review and editing. **Jennifer Gerdts**: Writing—review and editing. **Jing Zhao**: Writing—review and editing. **Josefine Gradman**: Writing—review and editing. **Julia E. M. Upton**: Writing—review and editing. **Julie Wang**: Writing—review and editing. **Kati Palosuo**: Writing—review and editing. **Kirsi M. Jarvinen**: Writing—review and editing. **Kirsten Beyer**: Writing—review and editing. **Kunling Shen**: Writing—review and editing. **Laura Polloni**: Writing—review and editing. **Lianne Mandelbaum**: Writing—review and editing. **Luciana Kase Tanno**: Writing—review and editing. **Lucy A. Bilaver**: Writing—review and editing. **Marcus S. Shaker**: Writing—review and editing. **Margitta Worm**: Writing—review and editing. **Maria Said**: Writing—review and editing. **Mary Kelly**: Writing—review and editing. **Mary Jane Marchisotto**: Writing—review and editing. **Michael Makris**: Writing—review and editing. **Mikaela Odemyr**: Writing—review and editing. **Montserrat Fernandez‐Rivas**: Writing—review and editing. **Motohiro Ebisawa**: Writing—review and editing. **Nandinee Patel**: Writing—review and editing. **Pablo Rodríguez del Río**: Writing—review and editing. **Pakit Vichyanond**: Writing—review and editing. **Paul Turner**: Writing—review and editing. **Pete Smith**: Writing—review and editing. **Pilar Morón Gaspar**: Writing—review and editing. **R. Sharon Chinthrajah**: Writing—review and editing. **Rima Rachid**: Writing—review and editing. **Roberta Bonaguro**: Writing—review and editing. **Ruchi Gupta**: Writing—review and editing. **Sabine Schnadt**: Writing—review and editing. **Sakura Sato**: Writing—review and editing. **Stefania Arasi**: Writing—review and editing. **Stephanie Leonard**: Writing—review and editing. **Sung Poblete**: Writing—review and editing. **Susanne Halken**: Writing—review and editing. **Thuy‐My Le**: Writing—review and editing. **Torsten Zuberbier**: Writing—review and editing. **Tracey Dunn**: Writing—review and editing. **Victoria Cardona**: Writing—review and editing.

## CONFLICT OF INTEREST STATEMENT

Antoine Deschildre declared consulting fees from Novartis, GSK, Sanofi, Regeneron, AstraZeneca, Aimmune Therapeutics, Stallergenes Greer, ALK, Viatris, Celltryon; advisory board for BOOM study NCT04045301, all outside the submitted work. Montserrat Alvaro Lozano declared no interests. Antonella Muraro declared speaker fees for Aimmune, DBV Technologies, Nestlè Health Science, Nestlè Purina; advisory boards for Aimmune, Sanofi, DBV Technologies, Novartis, Regeneron, Viatris, all outside the submitted work. Marcia Podesta declared institutional grants from Viatris, DBV Technologies, Novartis, Aimmune; honoraria from DBV Technologies; advisory board for Novartis; president Food Allergy Italia, member GA2LEN ANACare Patient Advocates team, board member EAACI Patient Organisation Committee, member EAACI ethics committee, member FAO WHO Codex Alimentarius Commission. Member EAACI task forces and guideline groups, all outside the submitted work. Debra de Silva declared institutional funds from GA²LEN towards drafting the manuscript. Mattia Giovannini reports personal fees from Sanofi, outside the submitted work. Simona Barni declared speaker fees from Nutricia, Sanofi, all outside the submitted work. Timothy E. Dribin declared grants from the National Institutes of Health, outside the submitted work. Mónica Sandoval‐Ruballos declared no interests. Aikaterini Anagnostou declared institutional grants from Novartis, AAFA, Mike Hogg Foundation; advisory boards for Ready Set Food, Novartis, Genentech, Bryn and consulting/speaker fees ALK, EPG Health, MJH, Adelphi, Aimmune Therapeutics, Genentech, FARE, Medscape, Innovation horizons, all outside the submitted work. Alessandro Fiocchi declared grants from Novartis, Ferrero, Sanofi, Stallergenes, Danone, Aimmune; consulting fees or honoraria from Abbott, Ferrero, Sanofi; board or leadership positions at World Allergy Organisation, American Academy of Allergy Asthema and Immunology, all outside submitted work. Alice Toniolo declared no interests. Andrew Bird declared institutional grants from Aimmune, Astellas, DBV Technologies, FARE, Genentech, NIH NIAID, Novartis, Regeneron and Siolta; consulting from Allakos, AllerGenis, Allergy Therapeutics, DBV Technologies, FARE, Genentech, HAL Allergy, Novartis, Nutricia and Parexel; speaking fees from DBV Technologies; data monitoring board for Infinant Health, all outside the submitted work. Angel Sánchez Sanz declared institutional payments for speaker fees from Aimmune Therapeutics and Advisory Board for Novartis Pharma AG. Anna Asarnoj declared grants/nn‐commercial contracts from Karolinska Institutet, Swedish Asthma and Allergy Association’s Research Foundation, Swedish Association for Allergology, Swedish Society of Medicine, speaker fees from Orion Pharma, Nestlé, Aimmune Therapeutics, Semper, ThermoFisher, Scandinavian Biopharma, ALK; peer review fees for PHRGOC24jan‐0004 Singapore; advisory board for Novartis, Sanofi, Danone, Nestlé, ALK; unpaid board member for SFFA, Research Council for the Foundation for Child Research, editorial board Pediatrician, education committee of Swedish Peediatric Society, all outside the submitted work. Anna Nowak‐Wegrzyn declared board roles at AAAAI, IFPIES, all outside the submitted work. Berber Vlieg‐Boerstra declared research grant from Nutricia Research; speaker fees or honoraria from Nestle, Nutricia; advisory board for Vinimini, all outside the submitted work. Brian P. Vickery declared institutional grants/contracts from Aimmune, Alladapt, Aravax, AstraZeneca, DBV Technologies, FARE , Genentech, Novartis, NIH‐NIAID, Regeneron, Siolta; consulting fees from Aimmune, Allergenis, Allakos, Aravax, Genentech, IgGenix, Novartis, Parexel, Reacta, Regeneron, Revolo, Sanofi, Sattergenes Greer; stock in Moonlight Therapeutics, all outside the submitted work. Carina Venter declared grant from Reckitt, speaker fees for Reckitt, Danone, Abbott, Nestle Nutrition Institute, all outside the submitted work. Caroline Nilsson declared institutional grant from Aimmune; institutional equipment from Thermofisher, honoraria to institution from ALK, GSK, Thermofisher; advisory board Aimmune, all outside the submitted work. Cecila Parente declared no interests. Céline Demoulin declared institutional grants or contracts from Aimmune, ALK, Bioprojet, DBV Technologies. Stallergenes Greer, Novartis, Thermo Fisher, Viatris Sante, all outside the submitted work. David M Fleischer declared institutional funding from ARS Pharmaceuticals, DBV Technologies; consultancy fees from Aquestive, ARS Pharmaceuticals, Byrn Pharma, DBV Technologies Genentech, Nasus; speaker fees from Genentech; royalties from UpTodate; stock options from Grow Happy, all outside the submitted work. Diola Bijhout declared no interests. Edward F. Knol declared no interests. Eleanor Garrow declared no interests. Emma E. Cook declared grant from JSPS Grant‐in‐Aid for Scientific Research; institutional consulting for Novartis Global Food Allergy Patient Council, outside the submitted work. Fallon Shulz declared no interests. Francesca Lazzarotto declared no interests. Francesca Mori declared consulting fees from ALK‐Abello, Aboca, all outside the submitted work. Gideon Lack declared institutional grants from National Institute of Allergy and Infectious Diseases, National Peanut Board; consulting fees from Novartis, DBV Technologies, Reckitt Mead Johnson, ALK Abello; speaker fees from DBV Technologies, Aimmune, EPG Health; stock in DBV Technologies, Mighty MissionMe, all outside the submitted work. Graham Roberts declared grants from NIHR, Food Standards Agency, Action medical Research, NIH; honoraria from ALK‐Abello, ThermoFisher; past president of BSACI, all outside the submitted work. Guillaume Pouessel declared consulting fees from Bioprojet, Theravia, Viatris, all outside the submitted work. Gustavo Andres Marino declared no interests. Helen Brough declared institutional research grants from National Institute of Allergy and Infectious Disease, DBV Technologies, Aimmune; advisory boards for Consortium for Food Allergy Research, National Institute for Allergy and Infectious Disease, National Institute for Health and Care Research, consultancy/speaker fees from DBV Technologies, Viatris, Parexel, Stallergenes, all outside the submitted work. Hugh A. Sampson declared institutional grants from NIAID, NIH, FARE; royalties from Wiley; consulting fees/honoraria from AAAAi, DBV Technologies, FARE, N‐Fold LLC, Alpina Biotech; advisory board for Siolta Therapeutics, FARE; stock from DBV Technologies, N‐Fold LLC all outside the submitted work. Jay Lieberman declared consulting fees from ARS, Aquestive, Bryn; honoraria from Medscape; leadership role for Joint Task Force for Practice Parameters, American Board of Allergy and Immunology, all outside the submitted work. Jennifer Gerdts declared institutional grants from National Peanut Board, Pfizer Canada, all outside the submitted work. Jing Zhao declared no interests. Josefine Gradman declared no conflict of interest. Julia E. M. Upton declared institutional grants from DBV Technologies, Alk‐Abello; institutional equipment from Novartis; consulting fees/honoraria from Pharming N.V, Pfizer, Viatris, Astra Zeneca, Bausch Health; member of Health Care Advisory Board Food Allergy Canada, editorial board Allergy, editorial board Annals of Allergy Asthma and Immunology; unpaid leadership role at CSACI, all outside the submitted work. Julie Wang declared institutional grants from Aimmune, DBV, Siolta, NIH; royalties from UpToDate; consulting fees from DBV, Novartis, all outside the submitted work. Kati Palosuo declared no interests. Kirsi M Järvinen declared grants to institution from NiAID, Janssen R&D, Aimmune, royalties from Wolter Klower, consulting fees from Harmony / Milk Care, Janseen R&D, speaker fees from AAAAI, all outside the submitted work. Kirsten Beyer declared institutional grants from Danone, DBV, Hipp, Infectopharm, Hycor, Novartis; advisory boards for Aimmune, Danone, Mylan, Nestle, Allergy Therapeutics, Novartis, Kantar Health, Primus Consulting; consultation/speaker fees from Hipp, Novartis, Aimmune, ALK, Danone, Infectopharm, Nestle, Hycor, ThermoFisher, Limbach Gruppe, Kantar Health, Sonic Heathcare; unpaid leadership or committee roles at GPA, DGAKI, DAAB, AGATE, NORA, all outside the submitted work. Kunling Shen declared no interests. Laura Polloni declared no interests. Lianne Mandelbaum declared no interests. Luciana Kase Tanno declared Director of the WHO Collaborating Centre on Classification Scientific Support and board member of WHO Medical and Scientific Advisory Committee Board, World Allergy Organisation and French Allergy Society, all outside the submitted work. Lucy A Bilaver declared institutional grants from NIH, Genentech, Novartis, Yobee Care, Northwestern University NUCATS, all outside the submitted work. Marcus Shaker declared member of Joint Task Force on Practice Parameters; editorial board of Journal of Allergy and Clinical Immunology In Practice; associate editor of Annals of Allergy, Asthma, and Immunology; Board of Directors of American Academy of Allergy, Asthma, and Immunology, all outside the submitted work. Margitta Worm declared honoraria/consulting fees from AbbVie, Aimmune Therapeutics, ALK‐Abelló, Allergopharma, Almirall, Amgen, AstraZeneca, Biotest, Boehringer Ingelheim, Bristol‐Myers Squibb, DBV Technologies, GlaxoSmithKline, Genzyme, Kymab, LEO Pharma, Lilly, Mylan, Novartis, Pfizer, Regeneron Pharmaceuticals Inc., Sanofi‐Aventis, Stallergenes Greer, Worg Pharmaceutics, all outside the submitted work. Maria Said declared no interests. Mary Jane Marchisotto declared no interests. Mary Kelly declared no interests. Michael Makris declared honoraria from Astra Zeneca, GSK, Novartis, Pfizer, Sanofi‐Aventis; Monitoring Board for Pfizer, Astra Zeneca, GSK, all outside the submitted work. Mikaela Odemyr declared no interests. Montserrat Fernandez‐Rivas declared institutional grants from Instituto de Salud Carlos III (Spanish Goverment), Diater, Aimmune Therapeutics, Novartis; speaker fees from EPG Health, HAL Allergy GSK; consulting fees from DBV, Aimmune Therapeutics, Novartis, Reacta Healthcare, SPRIM, Viatris, all outside the submitted work. Motohiro Ebisawa declared no interests. Nandinee Patel declared no interests. Pablo Rodríguez del Río declared research grants from FAES, Aimmune Therapeutics; speaker fees from DBV, GSK, FAES, Novartis, ALK‐Abelló, LETI Pharma, Aimmune Therapeutics, Sanofi, Stallergenes; consultancy fees from FAES, Miravo, all outside the submitted work. Pakit Vichyanond declared no interests. Paul Turner declared grants from UK Medical Research Council, NIHR‐Imperial Biomedical Research Centre, UK Food Standards Agency, Jon Moulton Charity Trust; honoraria from UK Food Standards Agency, all outside the submitted work. Pete Smith declared grant from Viatris, GSK, Sanofi; speaker fees from Bayer, Danone, Viatris, GSK, AZ, Sanofi, Mundipharama, Nutricia; board for Nestle Nutrition Institute, all outside the submitted work. Pilar Moron Gaspar declared no interests. R. Sharon Chinthrajah declared grants from the Consortium for Food Allergy Research (CoFAR), National Institute of Allergy and Infectious Disease (NIAID), Food Allergy Research & Education (FARE); advisory board member for Alladapt Immunotherapeutics, Novartis, Allergenis, Intrommune Therapeutics, Phylaxis, Latitude, Genentech, all outside the submitted work. Rima Rachid declared grants from Novartis, Siolta Therapeutics, NIAID CoFAR, Food Allergy Fund; speakers fees / honoraria from Eli Lilly, Genentech, Novartis, Medscape, FARE, EFAAC, MAAC; patents US patent application PCT/US2016/060353, US patent application: 62/823,866, US patent application 62/758,161(62/798,224), US patent application 17/801,238; Vice Chair, Food Allergy and Eosinophilic disorders interest section, AAAAI, all outside the submitted work. Roberta Bonaguro declared no interests. Ruchi Gupta declared institutional funding from NIH, FARE, Melchioree Family Foundation, Sunshine Charitable Foundation, Genentech, Novartis; consulting fees from FARE, Genentech, Novartis, OWYN, Kaleo, Aquestive Therapies, Bryn Pharma, patents with Yobee U.S. Patent No. 11,103,544, ownership interest in Yobee Care Inc. Sabine Schnadt declared speaker fees from Aimmune Therapeutics, DBV Technologies, Mylan Germany, all outside the submitted work. Sakura Sato declared no interests. Stefania Arasi declared speaker/consultancy fees from Ulrich, DBV, Stallergenes Greer, Thermofischer Scientific; monitoring board for Novartis, Stallergenes Greer, all outside the submitted work. Stephanie Leonard declared institutional grants from Aimmune Therapeutics, DBV Technologies, Siolta Therapeutics, Stanford University; past employment, consulting fees and stock options from Hanimune Therapeutics; honoraria from American College of Allergy, Asthma and Immunology, San Diego Allergy Society, Washington State Society of Allergy, Asthma, and Immunology; advisory board Cour Pharmaceuticals Development Co, all outside submitted work. Sung Poblete declared no interests. Susanne Halken declared speaker fees from ALK‐Abello, Nestlé Purino, Abigo, Meadjohnson; monitoring committee for Stallergenes, all outside the submitted work. Thuy‐My Le declared grants from Dutch Research Council and Novartis; honariaria from Abbvie, Thermofisher Scientific, Abbvie, all outside the submitted work. Torsten Zuberbier declared grants from Novartis and Henkel, consulting fees from Bayer Health Care, FAES, Novartis, Henkel, speaker fees from AstraZeneca, AbbVie, ALK, Almirall, Astellas, Blueprint, Beyer Health care, Baiersdorf, Bencard, Berlin Chemie, FAES, HAL, Henkel, Kryolan, Leti, L’Oreal, Meda, Menarini, Merck, MSD, Novartis, Pfizer, Sanofi, Stallergenes, Takeda, Teva and UCB; Board member or lead of DGAKI and ECARF, all outside the submitted work. President of GA2LEN. Tracey Dunn declared no interests. Victoria Cardona declared no interests.

## Supporting information

Supporting Information S1

## Data Availability

Data sharing not applicable to this article as no datasets were generated or analysed during the current study.

## References

[1] Spolidoro GCI , Amera YT , Ali MM , et al. Frequency of food allergy in Europe: an updated systematic review and meta‐analysis. Allergy. 2023;78(2):351‐368. 10.1111/all.15560 36271775 PMC10099188

[2] Warren CM , Sehgal S , Sicherer SH , Gupta RS . Epidemiology and the growing epidemic of food allergy in children and adults across the globe. Curr Allergy Asthma Rep. 2024;24(3):95‐106. 10.1007/s11882-023-01120-y 38214821

[3] Rona RJ , Keil T , Summers C , et al. The prevalence of food allergy: a meta‐analysis. J Allergy Clin Immunol. 2007;120(3):638‐646. 10.1016/j.jaci.2007.05.026 17628647

[4] Turner PJ , Campbell DE , Motosue MS , Campbell RL . Global trends in anaphylaxis epidemiology and clinical implications. J Allergy Clin Immunol Pract. 2020;8(4):1169‐1176. 10.1016/j.jaip.2019.11.027 31786255 PMC7152797

[5] Waserman S , Cruickshank H , Hildebrand KJ , et al. Prevention and management of allergic reactions to food in child care centers and schools: practice guidelines. J Allergy Clin Immunol. 2021;147(5):1561‐1578. 10.1016/j.jaci.2021.01.034 33965093

[6] Prosty C , Colli MD , Gabrielli S , et al. Impact of reaction setting on the management, severity, and outcome of pediatric food‐induced anaphylaxis: a cross‐sectional study. J Allergy Clin Immunol Pract. 2022;10(12):3163‐3171. 10.1016/j.jaip.2022.09.015 36162798

[7] Alvarez‐Perea A , Tomás‐Pérez M , Martínez‐Lezcano P , et al. Anaphylaxis in adolescent/adult patients treated in the emergency department: differences between initial impressions and the definitive diagnosis. J Investig Allergol Clin Immunol. 2015;25(4):288‐294. 26310044

[8] Pouessel G , Beaudouin E , Tanno LK , et al. Food‐related anaphylaxis fatalities: analysis of the allergy vigilance Network® database. Allergy. 2019;74(6):1193‐1196. 10.1111/all.13717 30636053

[9] Turner PJ , Gowland MH , Sharma V , et al. Increase in anaphylaxis‐related hospitalizations but no increase in fatalities: an analysis of United Kingdom national anaphylaxis data, 1992‐2012. J Allergy Clin Immunol. 2015;135(4):956‐963.e1. 10.1016/j.jaci.2014.10.021 25468198 PMC4382330

[10] Hui JW , Copeland M , Lanser BJ . Food allergy management at school in the era of immunotherapy. Curr Allergy Asthma Rep. 2020;20(8):32. 10.1007/s11882-020-00933-5 32506300

[11] Dupuis R , Kinsey EW , Spergel JM , et al. Food allergy management at school. J Sch Health. 2020;90(5):395‐406. 10.1111/josh.12885 32124441

[12] Tse Y , Rylance G . Emergency management of anaphylaxis in children and young people: new guidance from the Resuscitation Council (UK). Arch Dis Child Educ Pract Ed. 2009;94(4):97‐101. 10.1136/adc.2007.120378 19654399

[13] Shaker MS , Wallace DV , Golden DBK , et al. Anaphylaxis ‐ a 2020 practice parameter update, systematic review, and Grading of Recommendations, Assessment, Development and Evaluation (GRADE) analysis. J Allergy Clin Immunol. 2020;145(4):1082‐1123. 10.1016/j.jaci.2020.01.017 32001253

[14] Russell AF , Bingemann TA , Cooke AT , et al. The need for required stock epinephrine in all schools: a work group report of the AAAAI Adverse Reactions to Foods Committee. J Allergy Clin Immunol Pract. 2023;11(4):1068‐1082.e1. 10.1016/j.jaip.2022.12.047 36716997

[15] US Centers for Disease Control and Prevention (CDC) . Voluntary Guidelines for Managing Food Allergies in Schools and Early Care and Education Programs. US Department of Health and Human Services; 2013.

[16] Muraro A , de Silva D , Podesta M , et al. 10 Practical Priorities to Prevent and Manage Serious Allergic Reactions: GA2LEN ANACare and EFA Anaphylaxis Manifesto. CTA; 2024. (in press). 10.1002/clt2.70009 PMC11606857 39614094

[17] Muraro A , Clark A , Beyer K , et al. The management of the allergic child at school: EAACI/GA2LEN Task Force on the allergic child at school. Allergy. 2010;65(6):681‐689. 10.1111/j.1398-9995.2010.02343.x 20345502

[18] Muraro A , Worm M , Alviani C , et al. EAACI guidelines: anaphylaxis (2021 update). Allergy. 2022;77(2):357‐377. 10.1111/all.15032 34343358

[19] Vale S , Smith J , Said M , Mullins RJ , Loh R . ASCIA guidelines for prevention of anaphylaxis in schools, pre‐schools and childcare: 2015 update. J Paediatr Child Health. 2015;51(10):949‐954. 10.1111/jpc.12962 26428419

[20] Pouessel G , Dumond P , Liabeuf V , et al. Gaps in the management of food‐induced anaphylaxis reactions at school. Pediatr Allergy Immunol. 2019;30(7):767‐770. 10.1111/pai.13091 31172595

[21] Madooh L , Allahou S , Alshallal H , et al. Food allergy knowledge, attitudes and beliefs of kindergarten teachers in Kuwait: a cross‐sectional study. BMJ Paediatr Open. 2023;7(1):e001795. 10.1136/bmjpo-2022-001795 PMC10008333 36882233

[22] Ozturk Haney M , Ozbıcakcı S , Karadağ G . Turkish teachers' self‐efficacy to manage food allergy and anaphylaxis: a psychometric testing study. Allergol Immunopathol. 2019;47(6):558‐563. 10.1016/j.aller.2019.03.002 31174852

[23] Santos MJL , Merrill KA , Gerdts JD , Ben‐Shoshan M , Protudjer JLP . Food allergy education and management in schools: a scoping review on current practices and gaps. Nutrients. 2022;14(4):732. 10.3390/nu14040732 35215382 PMC8879822

[24] Ercan H , Ozen A , Karatepe H , Berber M , Cengizlier R . Primary school teachers' knowledge about and attitudes toward anaphylaxis. Pediatr Allergy Immunol. 2012;23(5):428‐432. 10.1111/j.1399-3038.2012.01307.x 22554351

[25] Rodríguez Ferran L , Gómez Tornero N , Cortés Álvarez N , Thorndike Piedra F . Anaphylaxis at school. Are we prepared? Could we improve? Allergol Immunopathol. 2020;48(4):384‐389. 10.1016/j.aller.2019.10.006 32061426

[26] Polloni L , Baldi I , Lazzarotto F , et al. School personnel's self‐efficacy in managing food allergy and anaphylaxis. Pediatr Allergy Immunol. 2016;27(4):356‐360. 10.1111/pai.12550 26887784

[27] Polloni L , Baldi I , Lazzarotto F , et al. Multidisciplinary education improves school personnel's self‐efficacy in managing food allergy and anaphylaxis. Pediatr Allergy Immunol. 2020;31(4):380‐387. 10.1111/pai.13212 31943386

[28] Polloni L , Lazzarotto F , Toniolo A , Ducolin G , Muraro A . What do school personnel know, think and feel about food allergies? Clin Transl Allergy. 2013;3(1):39. 10.1186/2045-7022-3-39 24274206 PMC4176479

[29] Tsuang A , Atal Z , Demain H , Patrick K , Pistiner M , Wang J . Benefits of school nurse training sessions for food allergy and anaphylaxis management. J Allergy Clin Immunol Pract. 2019;7(1):309‐311.e2. 10.1016/j.jaip.2018.05.015 29807143

[30] Sasaki K , Sugiura S , Matsui T , et al. A workshop with practical training for anaphylaxis management improves the self‐efficacy of school personnel. Allergol Int. 2015;64(2):156‐160. 10.1016/j.alit.2014.10.005 25838091

[31] Tsuang A , Menon NR , Bahri N , Geyman LS , Nowak‐Węgrzyn A . Risk factors for multiple epinephrine doses in food‐triggered anaphylaxis in children. Ann Allergy Asthma Immunol. 2018;121(4):469‐473. 10.1016/j.anai.2018.06.015 29940309

[32] Wahl A , Stephens H , Ruffo M , Jones AL . The evaluation of a food allergy and epinephrine autoinjector training program for personnel who care for children in schools and community settings. J Sch Nurs. 2015;31(2):91‐98. 10.1177/1059840514526889 24643758

[33] Higgs J , Styles K , Bowyer S , Warner A , Dunn Galvin A . Dissemination of EAACI Food Allergy Guidelines using a flexible, practical, whole school allergy awareness toolkit. Allergy. 2021;76(11):3479‐3488. 10.1111/all.14871 33894060 PMC8596703

[34] Pistiner M , Devore CD , Schoessler S . School food allergy and anaphylaxis management for the pediatrician. Extending the medical home with critical collaborations. Pediatr Clin. 2015;62(6):1425‐1439. 10.1016/j.pcl.2015.07.016 26456441

[35] Pouessel G , Turner PJ , Worm M , et al. Food‐induced fatal anaphylaxis: from epidemiological data to general prevention strategies. Clin Exp Allergy. 2018;48(12):1584‐1593. 10.1111/cea.13287 30288817

[36] Lloyd M , Loke P , Mack DP , et al. Varying approaches to management of ige‐mediated food allergy in children around the world. J Allergy Clin Immunol Pract. 2023;11(4):1010.e6‐1027.e6. 10.1016/j.jaip.2023.01.049 36805346

[37] Heideman K , Poronsky CB . Protocols for managing food allergies in elementary and secondary schools. Compr Child Adolesc Nurs. 2021;45(3):234‐246. 10.1080/24694193.2021.1883771 33792445

[38] Bartnikas LM , Huffaker MF , Sheehan WJ , et al. Impact of school peanut‐free policies on epinephrine administration. J Allergy Clin Immunol. 2017;140(2):465‐473. 10.1016/j.jaci.2017.01.040 28347736 PMC5546995

[39] McIntyre CL , Sheetz AH , Carroll CR , Young MC . Administration of epinephrine for life‐threatening allergic reactions in school settings. Pediatrics. 2005;116(5):1134‐1140. 10.1542/peds.2004-1475 16264000

[40] Ebisawa M , Ito K , Fujisawa T , et al. Japanese guidelines for food allergy 2020. Allergol Int. 2020;69(3):370‐386. 10.1016/j.alit.2020.03.004 33289637

[41] Cardona V , Ansotegui IJ , Ebisawa M , et al. World allergy organization anaphylaxis guidance 2020. World Allergy Organ J. 2020;13(10):100472. 10.1016/j.waojou.2020.100472 33204386 PMC7607509

[42] Tanno LK , Worm M , Ebisawa M , et al. Global disparities in availability of epinephrine auto‐injectors. World Allergy Organ J. 2023;16(10):100821. 10.1016/j.waojou.2023.100821 37915955 PMC10616381

[43] Muraro A , Mendoza Hernandez DA . Managing food allergy and anaphylaxis: a new model for an integrated approach. Allergol Int. 2020;69(1):19‐27. 10.1016/j.alit.2019.10.004 31759890

[44] Volerman A , Brindley C , Amerson N , Pressley T , Woolverton N . A national review of state laws for stock epinephrine in schools. J Sch Health. 2022;92(2):209‐222. 10.1111/josh.13119 34825371 PMC8884139

[45] Zadikoff EH , Whyte SA , Desantiago‐Cardenas L , Harvey‐Gintoft B , Gupta RS . The development and implementation of the Chicago public schools emergency EpiPen® policy. J Sch Health. 2014;84(5):342‐347. 10.1111/josh.12147 24707929

[46] Golden DBK , Wang J , Waserman S , et al. Anaphylaxis: a 2023 practice parameter update. Ann Allergy Asthma Immunol. 2024;132(2):124‐176. 10.1016/j.anai.2023.09.015 38108678

[47] Cicutto L , Julien B , Li N , et al. Comparing school environments with and without legislation for the prevention and management of anaphylaxis. Allergy. 2012;67(1):131‐137. 10.1111/j.1398-9995.2011.02721.x 21951319 PMC3237918

[48] Ford LS , Turner PJ , Campbell DE . Recommendations for the management of food allergies in a preschool/childcare setting and prevention of anaphylaxis. Expet Rev Clin Immunol. 2014;10(7):867‐874. 10.1586/1744666X.2014.914851 24773507

[49] Oxford MA , Hoyt AEW . The heterogeneity of stock epinephrine legislation in the United States. J Allergy Clin Immunol Pract. 2022;10(1):78‐80. 10.1016/j.jaip.2021.10.051 35000741

[50] Shaker MS , Greenhawt MJ . Analysis of value‐based costs of undesignated school stock epinephrine policies for peanut anaphylaxis. JAMA Pediatr. 2019;173(2):169‐175. 10.1001/jamapediatrics.2018.4275 30575857 PMC6439603

[51] Murdoch B , Adams EM , Caulfield T . The law of food allergy and accommodation in Canadian schools. Allergy Asthma Clin Immunol. 2018;14(1):67. 10.1186/s13223-018-0273-6 30377435 PMC6196567

[52] Pouessel G , Lejeune S , Dupond MP , Renard A , Fallot C , Deschildre A . Individual healthcare plan for allergic children at school: lessons from a 2015‐2016 school year survey. Pediatr Allergy Immunol. 2017;28(7):655‐660. 10.1111/pai.12795 28881055

[53] DeSantiago‐Cardenas L , Rivkina V , Whyte SA , Harvey‐Gintoft BC , Bunning BJ , Gupta RS . Emergency epinephrine use for food allergy reactions in Chicago Public Schools. Am J Prev Med. 2015;48(2):170‐173. 10.1016/j.amepre.2014.09.005 25442236

[54] Eldredge C , Patterson L , White B , Schellhase K . Assessing the readiness of a school system to adopt food allergy management guidelines. Wis Med J. 2014;113(4):155‐161. 25211803

[55] Raptis G , Perez‐Botella M , Totterdell R , Gerasimidis K , Michaelis LJ . A survey of school's preparedness for managing anaphylaxis in pupils with food allergy. Eur J Pediatr. 2020;179(10):1537‐1545. 10.1007/s00431-020-03645-0 32249360 PMC7479013

[56] https://allergy.org.au/images/stories/anaphylaxis/2025/ASCIA_Action_Plan_Anaphylaxis_General_2025.pdf (Accessed 18/11/2024).

[57] https://foodallergycanada.ca/tools‐and‐downloads/downloads/emergency‐plan‐forms/ (Accessed 6/11/2024).

[58] https://eduscol.education.fr/1207/poursuite‐de‐la‐scolarite‐avec‐des‐traitements‐medicaux‐particuliers (Accessed 18/11/2024).

[59] Ring J , Beyer K , Biedermann T , et al. Guideline (S2k) on acute therapy and management of anaphylaxis: 2021 update: S2k‐guideline of the German society for allergology and clinical immunology (DGAKI), the medical association of German allergologists (AeDA), the society of pediatric allergology and environmental medicine (GPA), the German Academy of allergology and environmental medicine (DAAU), the German professional association of pediatricians (BVKJ), the society for neonatology and pediatric intensive care (GNPI), the German society of dermatology (DDG), the Austrian society for allergology and immunology (OGAI), the Swiss society for allergy and immunology (SGAI), the German society of anaesthesiology and intensive care medicine (DGAI), the German society of pharmacology (DGP), the German respiratory society (DGP), the patient organization German allergy and Asthma association (DAAB), the German working group of anaphylaxis training and education (AGATE). Allergo J Int. 2021;30:1‐25. 10.1007/s40629-020-00158-y 33527068 PMC7841027

[60] https://www.foodallergyitalia.org/protocollo‐di‐emergenza/ (Accessed 6/11/2024).

[61] https://www.bsaci.org/wp‐content/uploads/2020/02/BSACIAllergyActionPlan2018NoAAI2981‐2.pdf (Accessed 6/11/2024).

[62] https://www.aaaai.org/aaaai/media/medialibrary/pdf%20documents/libraries/anaphylaxis‐emergency‐action‐plan.pdf (Accessed 6/11/2024).

[63] https://aap.org/aaep (Accessed 17/11/2024).

[64] Mustafa SS , Russell AF , Kagan O , et al. Parent perspectives on school food allergy policy. BMC Pediatr. 2018;18(1):164. 10.1186/s12887-018-1135-6 29753332 PMC5948763

[65] Polloni L , Gini G , Fiore G , et al. Bullying risk in students with food allergy: school teachers' awareness. Pediatr Allergy Immunol. 2016;27(2):225‐226. 10.1111/pai.12486 26360124

[66] Ross N , Dalke S , Filuk S , et al. It takes a village: perceptions of Winnipeg parents, students, teachers and school staff regarding the impact of food allergy on school‐age students and their families. Allergy Asthma Clin Immunol. 2022;18(1):47. 10.1186/s13223-022-00682-2 35689271 PMC9188203

[67] Waserman S , Shah A , Cruickshank H , Avilla E . Recognition and management of food allergy and anaphylaxis in the school and community setting. Immunol Allergy Clin. 2022;42(1):91‐103. 10.1016/j.iac.2021.09.008 34823753

[68] https://foodallergycanada.ca/professional‐resources/educators/school‐k‐to‐12/registration‐all‐about‐food‐allergy‐school‐program/ (Accessed November 14, 2024).

[69] Kao LM , Wang J , Kagan O , et al. School nurse perspectives on school policies for food allergy and anaphylaxis. Ann Allergy Asthma Immunol. 2018;120(3):304‐309. 10.1016/j.anai.2017.12.019 29508717

[70] Hogue SL , Muniz R , Herrem C , Silvia S , White MV . Barriers to the administration of epinephrine in schools. J Sch Health. 2018;88(5):396‐404. 10.1111/josh.12620 29609214

[71] Sicherer SH , Mahr T . Management of food allergy in the school setting. Pediatrics. 2010;126(6):1232‐1239. 10.1542/peds.2010-2575 21115583

[72] Dribin TE , Schnadower D , Wang J , et al. Anaphylaxis knowledge gaps and future research priorities: a consensus report. J Allergy Clin Immunol. 2022;149(3):999‐1009. 10.1016/j.jaci.2021.07.035 34390722 PMC8837706

[73] Dribin TE . The need to link anaphylaxis signs and symptoms with targeted therapeutic strategies. Ann Allergy Asthma Immunol. 2023;131(2):135‐136. 10.1016/j.anai.2023.05.024 37536870 PMC10696916

[74] Shaker M , Greenhawt M . Association of fatality risk with value‐based drug pricing of epinephrine autoinjectors for children with peanut allergy: a cost‐effectiveness analysis. JAMA Netw Open. 2018;1(7):e184728. 10.1001/jamanetworkopen.2018.4728 30646369 PMC6324395

[75] Shaker M , Greenhawt M . Cost‐effectiveness of stock epinephrine autoinjectors on commercial aircraft. J Allergy Clin Immunol Pract. 2019;7(7):2270‐2276. 10.1016/j.jaip.2019.04.029 31201119

