# Supplementary material for: Towards a common approach for managing food allergy and serious allergic reactions (anaphylaxis) at school. GA2LEN and EFA consensus statement
Source: Clin Transl Allergy. 2024 Dec 29;15(1):e70013. doi: 10.1002/clt2.70013 (PMC11682877; doi:10.1002/clt2.70013)
Supplement: Supplementary file 1 — Supporting Information S1 [file CLT2-15-e70013-s001.docx]

**Towards a common approach for managing food allergy and serious allergic reactions (anaphylaxis) at school. GA^2^LEN and EFA consensus statement.**

Supplementary information

This supplementary information describes feedback from patient organisations about the policies 21 countries have in place regionally or nationally to prevent and manage food allergy in schools. This document lists stakeholder perceptions of the local context. It is not a comprehensive picture. The aim is to show that there is a great deal of perceived variation between countries, rather than provide a detailed or precise picture of what happens in individual countries or provinces.

EFA and the working group for this article surveyed representatives from patient organisations and undertook desk-based research in summer 2023. Organisations from 21 out of 26 countries invited provided information, a response rate of 81%.

Participating organisations included:

- Argentina: SOS Alergia
- Australia: Allergy & Anaphylaxis Australia
- Brazil: Alergia Aliementar Brazil
- Canada: Food Allergy Canada
- Chile: Creciendo con Alergias
- China: Capital Institute of Pediatrics Affiliated Children Hospital
- Denmark: Asthma-Allergy Denmark
- France: French Allergy Prevention Association APFRAL
- Germany: German Allergy and Asthma Association DAAB
- Greece: personal contact
- Italy: Food Allergy Italia
- Japan: ATOPICCO Network for Children of the Earth
- Poland: Polish Federation of Asthma, Allergy and COPD Patients’ Organisations
- Portugal: personal contact
- Serbia: Allergy and Me
- Spain: Spanish Association for People with Food and Latex Allergy AEPNAA
- Sweden: Swedish Asthma and Allergy Association
- Switzerland: aha! Swiss Allergy Center; Allergissima
- Turkey: Living with Allergy Association
- UK: Allergy UK
- USA: Food Allergy & Anaphylaxis Connection Team FAACT; Food Allergy Research Education FARE

We asked patient organisations whether there were national or regional laws or mandates about each of these things:

| **Food allergy education programmes**   - Training for school staff and lunchroom staff about serious allergic reactions (anaphylaxis) - Adrenaline autoinjector training for school staff and lunchroom staff - Training for students - Educational material in the classroom relating to food allergy - Educational material in the lunchroom relating to food allergy |
| --- |
| **Lunchroom policies**   - School lunch menus with allergen information - Food items are labeled with allergen information - Clear cleaning procedures in the lunchroom - Designated lunch areas for students with food allergies, if appropriate |
| **Classroom policies**   - Guidelines for food in the classroom - Guidelines for food during celebrations (e.g. holidays and birthdays) |
| **Policies for field trips and after-school activities**   - Food policies for after-school activities - When the school does not provide food for field trips, all parents are provided with food guidelines |
| **Transportation to/from school**   - Policies for food if students take the school bus to/from school - Adult on the bus is trained about allergic reactions and using adrenaline autoinjector |
| **Adrenaline policies**   - Unassigned (stock) adrenaline is available in: - Kindergarten - Primary school - Middle-school - High-school - Students are able to carry their medications - A student’s adrenaline is readily available in the classroom and during school activities (e.g. sport) - Stock adrenaline available on school field trips - Stock adrenaline available for after-school activities - Stock adrenaline travels with groups outside of school - Schools have copies of individual Emergency Action Plan / letters from allergist |

The table below summarises key themes. **‘Shared policy’ means that there was some law or mandate shared across the country** or wide region. ‘No shared policy’ means there was no national or regional law or common approach reported, even though some schools and districts may follow shared principles. ‘No shared policy’ is not a positive or negative judgement because it may be more appropriate to have localised/regional policies than national laws depending on the local governance structure.

| **Country** | **Policies about training staff** | **Policies for place where students eat** | **Policies for food in classrooms** | **Policies for field trips and after sch activities** | **Policies for transport to and from school** | **Policies for adrenaline and emergencies** |
| --- | --- | --- | --- | --- | --- | --- |
| Argentina | No shared policy.  No national food allergy-specific policy. Allergist provides information to school staff. | No shared policy.  No shared policy specific to food allergy. Prepacked foods have allergen information. | No shared policy. | No shared policy. | No shared policy. | No shared policy.  Adrenaline autoinjectors are available in the country, but some schools do not feel comfortable storing and using. |
| Australia | Shared policies exist.  It is mandatory to train at least one staff member. Online training is recommended, such as twice-yearly briefings. Age-appropriate education is recommended and free resources are given to students and displayed. | Some shared policies.  It is recommended that age-appropriate risk minimisation strategies are put in place and that canteen staff complete food allergen management training for schools. Only packaged food is labelled. | Some shared policies.  Policy is to avoid blanket food bans.  If requested by primary schools or continuing education centres, peanuts and tree nuts may not be sent.. | Shared policies exist.  Mandatory that schools have a communication plan. | Some shared policies.  Strongly recommended to bring at least one adrenaline autoinjector. | Shared policies exist.  Unassigned adrenaline autoinjectors can be stocked (national policy). A site-specific risk assessment is advised to determine the number stocked. An emergency action plan is mandatory. |
| Brazil | Shared policies exist.  There is no shared policy specific to food allergy, but there is national law related to first aid in case of medical emergencies. | Some shared policies.  There is a national obligation to offer special meals in public schools. Prepacked foods have allergen information. There is no national policy for information on menus. There is no policy related to cleaning, but there is an obligation to have practices for handling food, which includes cleaning. | No shared policy. | No shared policy. | No shared policy. | No shared policy.  Some schools prohibit students from carrying their own medication, and some refuse to store and manage it. Autoinjectors are not for sale in Brazil. |
| Canada | Some shared policies.  Canada’s provinces and territories determine their own educational programmes for students and staff. Technically school staff should be trained, but there are no definitions for “staff” or “training”. | Some shared policies.  Some common procedures in elementary schools. Laws are set at provincial level, not national. In Canada most schools do not have a specific lunchroom. Students may eat in classrooms or outside. | Some shared policies.  Policies may differ by province, region, school district, school, and class. Policy usually aligns with policies for the rooms students eat. | No shared policy.  Policies may differ by province, region, school district, school, and class. Policy usually aligns with lunchroom policies. | No shared policy.  Policies may differ by province, region, school district, school, and class. Policy usually aligns with lunchroom policies. | Some shared policies.  The unassigned adrenaline policy is implemented nationally on voluntary basis. Many regions have at least one stock adrenaline device. Students are expected to bring autoinjectors when on field trips or offsite activities. Emergency action plan policies are implemented at regional level. |
| Chile | No shared policy.  Patient organisation provides trainers and educational materials reviewed by the Ministry of Health | No shared policy. | No shared policy. | No shared policy. | No shared policy. | No shared policy.  Most private schools allow students to carry autoinjectors. National emergency action plan template is available. |
| China | No shared policy. | No shared policy. | No shared policy. | No shared policy. | No shared policy. | No shared policy. |
| Denmark | No shared policy. | No shared policy.  All students bring their own lunches. No designated lunch areas for students with food allergies. | No shared policy. | No shared policy. | No shared policy. | No shared policy.  No unassigned adrenaline at kindergarten, primary school, middle-school or high-school. No policy about emergency action plans. |
| France | No shared policy. | Shared policies exist.  No designated lunch areas required. Menus required to have allergen info. | Shared policies exist. | Shared policies exist. | No shared policy. | Shared policies exist.  Schools store devices for all students prescribed adrenaline. Middle and high schools are allowed to store unassigned adrenaline. |
| Germany | Some shared policies.  Patient organisation, not government, provides free training.  Some content included in education for teachers about healthy nutrition. | Some shared policies.  No designated areas. School lunch menus must include allergen information.  Mandatory allergen information for non-prepacked food also applies to school meals.  Cleaning procedures in not mandated. | No shared policy. | No shared policy. | No shared policy. | No shared policy.  No unassigned adrenaline. If a student is prescribed an autoinjector some childcare centres and schools will store it.  Students are able to carry their medication depending on their age. Emergency action plan template available but not mandatory. |
| Greece | No shared policy. | No shared policy. | No shared policy. | No shared policy. | No shared policy. | Shared policy exists.  No unassigned adrenaline is available. Most schools require students to keep autoinjectors at school. |
| Italy | Some shared policies.  Partially implemented at regional level. | Shared policy exists.  National guidelines on school catering with a chapter dedicated to food allergy and national policies banning the introduction and consumption of food for collective use in schools. | Shared policy exists. | Shared policy exists.  Food policies are applicable for both school and after-school activities. | Some shared policies..  National legislation exists but school transportation depends on the city, provinces and local regulation. | Some shared policies.  No unassigned adrenaline is available. Most schools require students to keep autoinjectors at school.  Emergency action plan template is available but not mandated. Letter from allergist is mandatory. |

| **Country** | **Policies about training staff** | **Policies for place where students eat** | **Policies for food in classrooms** | **Policies for field trips and after sch activities** | **Policies for transport to and from school** | **Policies for adrenaline and emergencies** |
| --- | --- | --- | --- | --- | --- | --- |
| Japan | Shared policy exists.  Training required for school staff but people who make lunches do not attend the same training. Food allergy is included in the guidelines about lunch, but not necessarily discussed in the lunchroom. | Some shared policies.  Most schools have different colour plates to illustrate if a person has food allergies. Some high schools have lunchrooms but most younger children eat in the classroom. Students typically do the cleaning. There are cleaning procedures at facilities that make school lunches. | Some shared policies.  Policies agreed at kindergarten but not at other levels. | No shared policy. | No shared policy. | Some shared policies.  Unassigned adrenaline is not available. By law can only use personal medicines. |
| Poland | Some shared policies.  Educational plan coproduced by stakeholder organisations. Piloted in primary school. | No shared policy. | No shared policy. | No shared policy. | No shared policy. | Some shared policies.  Unassigned adrenaline not available. Students are able to carry their medications and national advice about having them available during school activities. Guidance on emergency action plans published but not implemented. |
| Portugal | No shared policy. | No shared policy. | No shared policy. | No shared policy. | No shared policy. | Some shared policies.  Students are able to carry their medications. Unassigned adrenaline available in schools larger than 1000 students. |

| **Country** | **Policies about training staff** | **Policies for place where students eat** | **Policies for food in classrooms** | **Policies for field trips and after sch activities** | **Policies for transport to and from school** | **Policies for adrenaline and emergencies** |
| --- | --- | --- | --- | --- | --- | --- |
| Serbia | No shared policy. | No shared policy.  Most kindergartens and schools do not have meals for students with food allergies. | No shared policy.  No guidelines for classroom or celebrations apart from in kindergarten in capital city. | No shared policy.  When food is not provided for the school trip caregivers either provide the food or the student is excluded from the trip. | No shared policy. | Some shared policies.  Unassigned adrenaline not available. Students are able to carry their medications with a doctor’s report. |
| Spain | No shared policy. | Some shared policies.  School menus must consider allergens and be adapted to the needs of students with food allergies. The student can bring their own food. By law, schools must provide a gluten-free menu and other accommodations when requested if supported by a medical certificate but this is not required for allergy sufferers | No shared policy. | No shared policy. | No shared policy. | No shared policy. |
| Sweden | No shared policy. | Some shared policies.  In many schools in Sweden nuts and peanuts are not allowed. Food is expected to be labelled. | Some shared policies. | No shared policy. | No shared policy. | Some shared policies.  Adrenaline is only available if it is prescribed to the student. If there is a trip or an activity outside school the student’s adrenaline should always be brought. |

| **Country** | **Policies about training staff** | **Policies for place where students eat** | **Policies for food in classrooms** | **Policies for field trips and after sch activities** | **Policies for transport to and from school** | **Policies for adrenaline and emergencies** |
| --- | --- | --- | --- | --- | --- | --- |
| Switzerland | No shared policy.  Training is offered but not mandatory. Educational material is available for classrooms and lunchrooms but it is not mandatory, | Some shared policies.  Catering staff provide allergen information. Hygiene management is included in guidelines. | No shared policy. | No shared policy. | No shared policy. | Shared policies exist.  Federal and regional rules exist about prescribing adrenaline to keep with students. In anaphylaxis the patient’s adrenaline can be used if the patient is conscious. This is expected to be the school nurse. Emergency action plan and allergist letters are used. |
| Turkey | Some shared policies.  An official education programme has begun. The Ministry of Education is designing an online education programme for all teachers across the country. | Some shared policies.  Not all government schools are full day so some do not have lunch service. Many schools have separate seating areas. It is mandatory to mention allergens on school menus. Usually a teacher is assigned to accompany the allergic student’s lunch table. | No shared policy. | No shared policy. | No shared policy.  Most students travel to school with parents or walk themselves. If not, parents inform the school bus staff. Schools usually do not have their own buses. | Some shared policies.  Stocking unassigned adrenaline is allowed but not mandatory. Students provide autoinjectors to store at school. A policy is being developed for stocking unassigned adrenaline and emergency action plans. |

| **Country** | **Policies about training staff** | **Policies for place where students eat** | **Policies for food in classrooms** | **Policies for field trips and after sch activities** | **Policies for transport to and from school** | **Policies for adrenaline and emergencies** |
| --- | --- | --- | --- | --- | --- | --- |
| UK | Shared policies exist.  Basic first aid training includes anaphylaxis. Autoinjector training is provided for school staff and lunchroom staff. | Some shared policies.  Scotland, Wales, Northern Ireland and England each have different policies. Prepacked foods are labelled. Loose / served food not labelled. Allergen information is available via local authority websites and can be pre-ordered after contact with the local authority (which runs schools). Shared cleaning policies. | No shared policy.  Teaching and support staff are made aware of students with food allergies and emergency action plans are used. However understanding is dependent on staff knowledge of allergy. | No shared policy.  Policies state that reasonable adjustments should be made for the student. Varies by school. | Some shared policies.  Guidelines in place but autoinjectors are to be administered only if carer / responsible adult feels competent to use them. | Shared policies exist.  Unassigned adrenaline is allowed to be stored in all settings apart from kindergarten. Legislation to enable schools to stock autoinjectors only applicable to primary, middle and high school. Most schools do not purchase autoinjectors due to cost. Students can carry their own medicine from age 11. This is available in the classroom and all school activities: Stock template for emergency action plan is available, with regional variations. |
| USA | Shared policies exist.  National recommendation that all school staff are trained about allergy and recommended to teach all students and their family members about food allergies | Shared policies exist.  Schools that participate in specific national meal programmes must accommodate students with ‘disabilities’ and provide them with a safe meal and a safe environment to consume the meal. Outside this, there are national recommendations about creating allergen-safe zones or food-free zones, providing advance copies of menus for parents to use in planning and to share ingredient labels, and cleaning system. | Shared policies exist.  National recommendation about designating allergen-safe zones and/or food-free zones, avoiding the use of identified allergens in the classroom and for class celebrations and using food-free items as prizes and rewards. | Some shared policies.  There is a national recommendation that meals and snacks are to be appropriately packaged to prevent cross-contact. | Shared policies exist.  The national recommendation is that all transportation staff receive training on responding to food allergy emergencies and administering adrenaline. | Shared policies exist.  The national recommendation is for schools to keep a dose of student’s adrenaline in an accessible location and for students to carry their own. There are financial incentives for states to pass laws that require junior and middle schools to have stock adrenaline. Laws vary by state, with some mandating stock adrenaline and some allowing. Emergency action plans are recommended. |
